# Profiling of the anti-malarial drug candidate SC83288 against artemisinins in *Plasmodium falciparum*

**DOI:** 10.1186/s12936-018-2279-4

**Published:** 2018-03-20

**Authors:** Maëlle Duffey, Cecilia P. Sanchez, Michael Lanzer

**Affiliations:** 1 Department of Infectious Diseases, Parasitology, Universitätsklinikum Heidelberg, Im Neuenheimer Feld 324, 69120 Heidelberg, Germany; 2 German Center for Infection Research (DZIF), Partner Site Heidelberg, 69120 Heidelberg, Germany

**Keywords:** *Plasmodium falciparum*, Anti-malarial drug, Artemisinin, Severe malaria, Drug development, Artemisinin-based combination therapy, Resistance

## Abstract

**Background:**

The increased resistance of the human malaria parasite *Plasmodium falciparum* to currently employed drugs creates an urgent call for novel anti-malarial drugs. Particularly, efforts should be devoted to developing fast-acting anti-malarial compounds in case clinical resistance increases to the first-line artemisinin-based combination therapy. SC83288, an amicarbalide derivative, is a clinical development candidate for the treatment of severe malaria. SC83288 is fast-acting and able to clear *P. falciparum* parasites at low nanomolar concentrations in vitro, as well as in a humanized SCID mouse model system in vivo. In this study, the antiplasmodial activity of SC83288 against artemisinins was profiled in order to assess its potential to replace, or be combined with, artemisinin derivatives.

**Results:**

Based on growth inhibition and ring survival assays, no cross-resistance was observed between artemisinins and SC83288, using parasite lines that were resistant to either one of these drugs. In addition, no synergistic or antagonistic interaction was observed between the two drugs. This study further confirmed that SC83288 is a fast acting drug in several independent assays. Combinations of SC83288 and artesunate maintained the rapid parasite killing activities of both components.

**Conclusion:**

The results obtained in this study are consistent with artemisinins and SC83288 having distinct modes of action and different mechanisms of resistance. This study further supports efforts to continue the clinical development of SC83288 against severe malaria as an alternative to artemisinins in areas critically affected by artemisinin-resistance. Considering its fast antiplasmodial activity, SC83288 could be combined with a slow-acting anti-malarial drug.

## Background

**Fig. 1:**
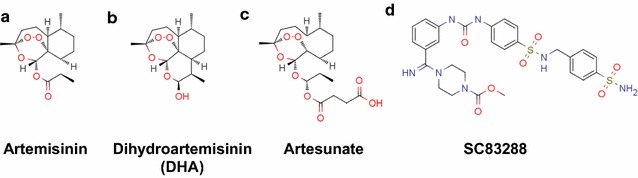
Structures of various antiplasmodial compounds. **a** artemisinin, **b** dihydroartemisinin, **c** artesunate, and **d** SC83288

Despite a decline in global malaria mortality during the previous decade, the human malaria parasite *Plasmodium falciparum* continues to infect more than 200 million people annually, causing approximately 450,000 deaths per year [1]. A main factor in the relative success in decreasing malaria-related deaths was the introduction of the highly efficient chemotherapeutic agent artemisinin (Fig. 1a) and its derivatives (Fig. 1b, c). Partnered with other drugs as artemisinin-based combination therapy (ACT), artemisinins have formed the backbone of malaria treatment and control since 2006 [2]. However, *P. falciparum* strains displaying delayed and possibly reduced responsiveness to ACT have emerged in western Cambodia [3], and are now spreading across all of Southeast Asia [4, 5]. Accordingly, there is a need for new anti-malarial drugs with novel chemical structures that exploit distinct molecular targets to protect them from cross-resistance mechanisms. While several clinical candidates fulfilling these criteria are currently in preclinical and clinical development [6–19], more candidates are needed to build up a solid and reliable reserve library of anti-malarial drugs should the first line drugs fail. One such clinical development candidates is SC83288 (Fig. 1d), which is considered for the treatment of severe malaria [20].

SC83288, an amicarbalide derivative, is active against *P. falciparum* blood stages at low nanomolar concentrations in vitro, with IC_50_ values of 3 nM. SC83288 is able to clear a *P. falciparum* infection in a humanized NOD/SCID mouse model system within 48 h following intraperitoneal injections of 2.5 mg kg^−1^ once per day over a period of 3 days [20]. Furthermore, SC83288 is fast acting and it has favourable pharmacological and toxicological features [20]. In vitro resistance to SC83288 develops slowly and might involve mutational changes in the Ca^2+^ transporting PfATP6, the *P. falciparum* SERCA orthologue. However, PfATP6 does not seem to be the molecular target of SC83288 but rather a component of resistance [20].

PfATP6 is a validated drug target [21] and was once considered the prime molecular target of artemisinins [22–25] and a contributor to artemisinin resistance [26–29]. However, recent studies failed to detect a specific interaction between PfATP6 and artemisinins [30–35], and instead favour a pleiotropic mode of action in that many proteins are covalently modified by the alkylating property of artemisinins [36]. Moreover, resistance to artemisinins has recently been associated with polymorphisms in the kelch propeller domain protein 13 (PfK13) [37–46], a member of the kelch protein superfamily, bearing multiple protein–protein interaction sites and involved, among others, in ubiquitin-regulated protein degradation and oxidative stress responses [47].

Considering the gold standard status of artemisinin and its derivatives in the anti-malarial drug development field and taking into account that PfATP6 has repeatedly been considered a drug target of, or a contributor to drug resistance, including to SC83288, raises the possibility that SC83288 and artemisinins exert their antiplasmodial activity, or induce resistance, in *P. falciparum* in a similar or synergistic manner. The data presented herein suggest that these concerns are unfounded. No cross-resistance was observed between dihydroartemisinin and SC83288 in vitro. Moreover, both drugs showed neither a synergistic nor antagonistic interaction. Consistent with previous results, SC83288 was found to be a fast acting compound, although SC83288 is not as rapid regarding its in vitro speed of action as are artemisinins [20]. Furthermore, combining SC83288 with artesunate did not affect the in vitro speed of action of either drug, allowing for the possibility of a combination therapy.

## Methods

### Chemicals

The chemicals used in this study were obtained from the Roth, Merck, Sigma, Serva, Thermo and Applichem. SC83288 was provided by the 4SC AG, München, Germany.

### Parasite culture

*Plasmodium falciparum* parasites were maintained in continuous in vitro culture essentially as previously described [48]. Briefly, cultures were maintained at a haematocrit of 5.0% and at a parasitaemia of not higher than 5% (if not stated otherwise) in RPMI 1640 supplemented with 2 mM l-glutamine, 25 mM HEPES, 100 µM hypoxanthine, 20 µg ml^−1^ gentamycin, 5% human heat-inactivated AB-positive serum and 5% Albumax. Cultures were incubated at 37 °C under controlled atmospheric conditions of 5% O_2_, 3% CO_2_, 92% N_2_ and 96% humidity. Synchronization of cultures at the ring stage was performed using a 5% sorbitol solution as previously described [49].

### *Plasmodium falciparum* growth inhibition assay

Growth inhibition assays were performed according to a standardized protocol, based on the detection of parasite DNA by fluorescent SYBRGreen staining [50, 51]. Briefly, synchronized cultures of *P. falciparum* ring-stage parasites were incubated in the presence of increasing drug concentrations, at the following final condition: 100 µl per well, 0.5% parasitaemia, 2% haematocrit, 72 h incubation at 37 °C. After incubation, the plates were frozen at − 80 °C overnight. On the day of the measurement, plates were thawed for 1 h at room temperature. The plates were filled with 100 µl per well of a 1× SYBRGreen (ThermoFisher Scientific Inc.) solution in lysis buffer (H_2_0, 20 mM Tris base pH 7.4, 5 mM EDTA, 0.008% (w/v) Saponin, 0.08% (w/v) Triton X-100), briefly shaken and incubated at room temperature for 1 h. Plates were protected from direct light. Fluorescence was measured in a fluorescence plate reader (FluoStar Optima, BMG Labtech GmbH) (ext/em: 485/520 nm, gain 1380, 10 flashes/well, top optic). The IC_50_ values were calculated using a four parameter sigmoidal function (SigmaPlot 13, Systat Software Inc.). 6–13 independent biological replicates were performed, each with technical duplicates.

### Ring survival assay

The in vitro ring survival assay (RSA) rate was assessed as previously described [52]. Briefly, tightly synchronized early ring parasites (0–3 h post invasion) at a parasitaemia of 0.5–1% were exposed to various drugs at a concentration of 700 nM for 6 h. The drug was subsequently washed-out, and the parasites were returned to culture for 66 h before being collected. DNA content analysis was performed by flow cytometry on SYBRGreen-stained parasites to measure the precise parasitaemia, as previously described [53], using a BD FACS Canto. Briefly, the parasites were fixed in PBS 4% paraformaldehyde, 0.0075% glutaraldehyde for 2 h at room temperature and permeabilized in PBS 0.1% Triton-100 for 8 min. The samples were subsequently subjected to a light RNase treatment (0.3 mg/ml in PBS) for 30 min at 37 °C. DNA was stained with SYBRGreen (1:2000 dilution in PBS) for 20 min in the dark. Parasites were then transferred to tubes adapted for flow cytometry. The fluorescence signal was collected in the green channel (FITC-H) of the FACS Canto. A minimum of 2000 infected red blood cells were counted per measurement and plotted in the form of dot-plots, using the software FlowingSoftware2.5.1. Parasites survival rates [RSA score (%)] were expressed as a percentage, by comparing the parasitaemia between the drug-treated and the untreated control as followed:

$${\text{RSA score }}\left( \% \right) = {\text{P}}_{{{\text{drug}} - {\text{treated}}}} \left( \% \right)/\left( {{\text{P}}_{{{\text{PBS}} - {\text{treated}}}} \left( \% \right)} \right) \times 100.$$

3–6 independent biological replicates were performed, each with technical duplicates.

### Drug interaction study

The interaction profile was assessed using the *P. falciparum* strain Dd2 and the fixed-ratio isobologram method [54]. This approach is based on determining the IC_50_ values of two individual drugs alone and in combination at different concentration ratios. In principle, the shift of a drug’s IC_50_ value, assessed in combination compared to the one determined for this drug alone, is indicative of the nature of the interaction between the two drugs [54]. Briefly, a synchronous culture of ring-stage *P. falciparum* blood-stage parasites was incubated for 72 h in the presence of increasing drug concentrations in a 96-well black microtiter plate. The final conditions of the tests were 100 µl per well, 0.5% parasitaemia, 2% final haematocrit and 72 h incubation time at 37 °C. On the day of the assay, a synchronized ring-stage culture, was established at 0.5% parasitaemia and 4% haematocrit. A solution of uninfected erythrocytes at 4% haematocrit was used as a negative control. Drugs were applied alone (A or B) and in combination at fixed concentration ratios (A:B) and analysed in threefold serial dilutions. Drug mixtures were prepared from 2x working solutions of each drug in the following fixed concentration ratios (v/v): ratio A:B = 4:1, 3:2, 2.5:2.5, 2:3, 1:4, directly in the 96-well plate. IC_50_ values of drugs A and B alone were determined on the same plate to ensure accurate calculation of drug interactions. After 72 h of incubation, plates were wrapped in aluminum foil and frozen at − 80 °C overnight. Parasite growth was assessed using SYBRGreen as described above. Graphical analysis was performed on isobolograms, obtained by plotting pairs of fractional IC_50_ (FIC_50_) values of drug A and drug B for each combination. The in vitro interactions were evaluated based on graphical and arithmetical analysis. It is commonly accepted that a mean sum of fractional 50% inhibitory concentrations (mean ∑FIC_50_) lower than 0.5 corresponds to a synergistic interaction, and higher than 1.5 to an antagonistic interaction [55]. A mean ∑FIC_50_ between these two cut-off values represents an indifferent interaction between the two investigated drugs. 7 independent biological replicates were performed, each with technical duplicates.

### In vitro speed of action

Two independent methods were used to determine the in vitro speed of action of drugs. The relative speed of action was obtained following the approach previously described by Le Manach et al. [56]. Briefly, the growth of an asynchronous parasite blood culture in the presence of anti-malarial compounds was assessed using SYBRGreen staining and expressed as IC_50_ values, as described above. For each compound, three exposure times were employed 72 (standard assay time), 48 and 24 h. The IC_50_ ratios, normalized to the 72 h exposure were subsequently used to classify the compound as fast- or slow-acting. The absolute speed of action was obtained using the method described by Linnares et al. [57]. Briefly an asynchronous culture of *P. falciparum* Dd2 blood-stage parasites, at 0.5% parasitaemia and 4% haematocrit was treated with a concentration corresponding to 10 × IC_50_ for a duration of 8, 14, 24, 48 or 72 h, in a final volume of 100 µl per well. Drugs were refreshed every 24 h. After incubation, the parasites were washed-out in drug free medium and resuspended in 100 µl of medium. 70 µl were then transferred into a fresh plate, containing 130 µl of CFDA-stained uninfected erythrocytes at 2% haematocrit, resulting in a 1/3 dilution. After 48 h of subsequent incubation at 37 °C, the parasites were washed in PBS, and the DNA was stained using Draq5 (1:500 dilution in PBS) for 20 min at room temperature. The samples were washed once with PBS and fixed in PBS 0.05% glutaraldehyde for 1 h at room temperature. The fixed samples were kept in PBS at 4 °C until flow cytometry analysis, using a BD FACS Canto. Collecting fluorescence signals in the FITC and the PerCP-Cy5 channel allowed the proportion of the double stained infected erythrocytes to be determined, which corresponds to the new infections. Results were displayed in the form of dot-plots, using the software FlowingSoftware2.5.1. The total infections were calculated as followed:

$${\text{Total infections}} = 3/2*{\text{RBC}}^{{{\text{CFDA}}/{\text{Draq}}5}} .$$

The parasite viability was then expressed as a percentage of the untreated parasites. 4 independent biological replicates were performed, each with technical duplicates.

## Results

### SC83288 shows no cross-resistance with artemisinins

The matter of cross-resistance between SC83288 and artemisinins was initially addressed, using two distinct approaches. On the one hand, standard growth inhibition assays over 72 h of drug exposure were performed, and on the other hand, the susceptibility of early ring stages in a ring survival assay (RSA) was evaluated. The RSA takes into account the antiplasmodial activity of artemisinin and its derivatives against the early blood stages [36, 52]. Four different strains of *P. falciparum* were used in these assays: the artemisinin and SC83288 sensitive *P. falciparum* strains NF54 and Dd2, the genetically engineered artemisinin-resistant NF54 mutant line bearing a mutation in the *kelch 13* gene (PfK13 C580Y), an established marker of artemisinin resistance [52], and a Dd2 derived SC83288 resistant line [20]. The two drug resistant clones were termed NF54^ART^ and Dd2^SC^.

**Fig. 2:**
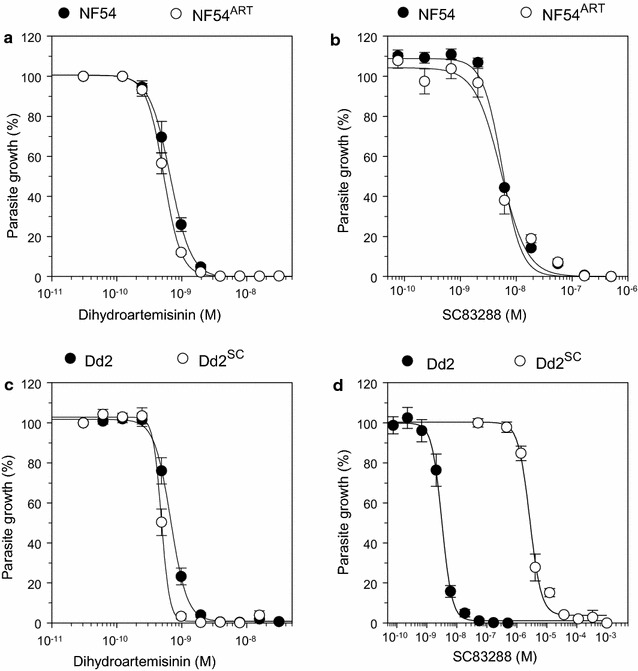
Susceptibility of different *P. falciparum* strains to SC83288 and dihydroartemisinin. **a** and **b** Growth inhibition of NF54 and the artemisinin resistant derivative NF54^ART^ carrying the PfK13 C580Y mutation by artemisinin (**a**) and SC83288 (**b**) in a standard cell proliferation assay with an exposure time of 72 h. **c** and **d** Growth inhibition of Dd2 and the SC83288 resistant derivative Dd2^SC^ by artemisinin (**c**) and SC83288 (**d**). Mean ± SEM of 6–13 independent determinations

In the growth inhibition assay, the wild-type NF54 line and the NF54^ART^ mutant displayed no significant difference in their dihydroartemisinin responsiveness, with IC_50_ value of 0.7 ± 0.1 and 0.5 ± 0.1 nM, respectively (Student’s t test, p = 0.9) (Table 1 and Fig. 2a). Both strains had also comparable susceptibilities to SC83288, with IC_50_ values of 5.1 ± 0.7 and 4.9 ± 0.8 nM, respectively (Student’s t test, p = 0.2) (Table 1 and Fig. 2b). In comparison, Dd2^SC^ and the parental Dd2 strain revealed comparable IC_50_ values to dihydroartemisinin (0.5 ± 0.1 and 0.7 ± 0.1 nM, respectively, Student’s t test, p = 0.8) (Table 1 and Fig. 2c) but different responses to SC83288 (1.0 ± 0.2 µM and 3.1 ± 0.2 nM, respectively, Student’s t test, p < 0.001) (Table 1 and Fig. 2d).

**Table 1:** Susceptibility of NF54, NF54^ART^, Dd2, and Dd2^SC^ to SC83288 and artemisinin

Strain	IC_50_ (nM)	
	SC83288	Dihydroartemisinin
NF54	5.1 ± 0.7	0.7 ± 0.1
NF54^ART^	4.9 ± 0.8	0.5 ± 0.1
Dd2	3.1 ± 0.2	0.7 ± 0.1
Dd2^SC^	1000 ± 200*	0.5 ± 0.5

*IC*_*50*_ inhibitory concentration of 50% of the maximal effect. Mean ± SEM of 6–13 independent biological replicates

*p < 0.001 as compared with the parental strain

**Fig. 3:**
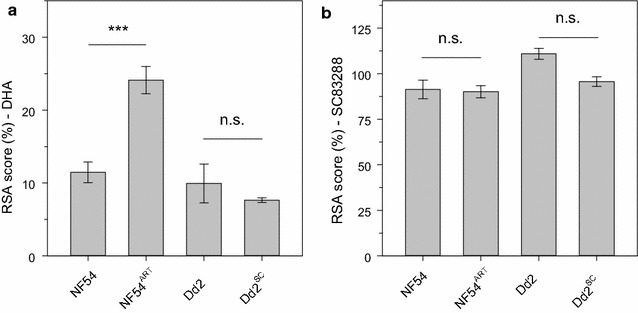
Ring stage susceptibility of different *P. falciparum* strains to dihydroartemisinin and SC83288. **a** Ring survival assay (RSA) scores of NF54, NF54^ART^, Dd2, and Dd2^SC^ to dihydroartemisinin (DHA). **b** RSA scores to SC83288. Mean ± SEM of 3–6 independent determinations. Statistical significance was evaluated using the one way ANOVA Holm-Sidak test. ***p < 0.001; *n.s.* not significant

Artemisinins have been shown to exert part of their antiplasmodial activity against very early ring-stages parasites, just after erythrocyte invasion [58, 59]. As expected [52], the NF54^ART^ line showed a significant difference in its RSA score to dihydroartemisinin (DHA) compared to the wild-type NF54 strain (RSA score of 11.5 ± 1.5 and 24.1 ± 1.9%, respectively, Student’s t test, p < 0.001). In comparison, Dd2 and Dd2^SC^ had low and comparable RSA scores of 9.9 ± 2.7 and 7.7 ± 0.3%, respectively (One-Way ANOVA Holm-Sidak test, p = 0.2), indicative of artemisinin susceptibility (Fig. 3a). Importantly, none of the parasites lines showed a ring-stage susceptibility to SC83288 (RSA score of 111.0 ± 3.0, 95.7 ± 2.7, 91.4 ± 5.1 and 90.1 ± 3.3%, for Dd2, Dd2^SC^, NF54, and NF54^ART^, respectively; One-Way ANOVA Holm-Sidak test, p = 0.8) (Fig. 3b), confirming the lack of activity of SC83288 against early ring-stages [20]. Taken together, the data from these two independent assays strongly suggest against any kind of cross-resistance effects between artemisinins and SC83288 in vitro.

### SC83288 and artemisinin do not interact

**Fig. 4:**
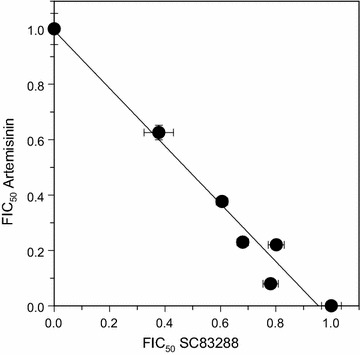
Indifferent interaction between artemisinin and SC83288. The fractional half-maximal inhibitory concentrations (FIC_50_) of artemisinin and SC83288 in the *P. falciparum* strain Dd2 were displayed in an isobologram. A linear regression was fitted to the data points. Mean ± SEM of 7 independent determinations

Subsequently, the question was addressed of whether artemisinin and SC83288 have an effect on one another when combined in a growth inhibition assay. To this end, the interaction profile between artemisinin and SC83288 using a fixed-ratio isobologram method [54] was investigated. A synchronous culture of Dd2 at the ring stage for 72 h was incubated in the presence of increasing drug concentrations, whereby artemisinin and SC83288 were applied alone and in combination at fixed concentration ratios. The arithmetical analysis revealed a mean sum of fractional 50% inhibitory concentrations (mean ∑FIC_50_) of 0.97 ± 0.06, suggestive of an indifferent interaction. The graphical analysis on the isobologram, obtained by plotting the pairs of fractional IC_50_ (FIC_50_) values of artemisinin and SC83288 for each combination, agreed with the arithmetical observation, following almost perfectly the diagonal trend line (fit to a linear polynomial curve with a R^2^ = 0.972) (Fig. 4), thereby corroborating the conclusion that both drugs do not interact.

### Combining SC83288 and artesunate does not affect the in vitro speed of action

**Fig. 5:**
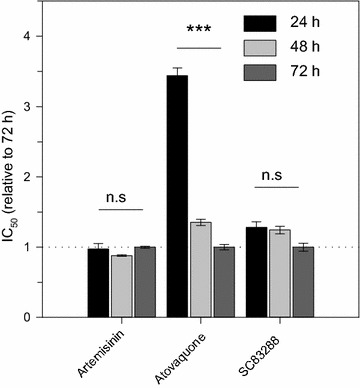
IC_50_-based relative speed of action profiles of SC83288, artemisinin, and atovaquone. The IC_50_ values of artemisinin, atovaquone and SC83288 were determined at exposure times of 24, 48 and 72 h in the *P. falciparum* Dd2 strain [56]. The data are shown normalized to the value obtained at the 72 h time point. Mean ± SEM of 6 independent determinations. Statistical significance was evaluated using the one way ANOVA Holm-Sidak test. ***p < 0.001; *n.s.* not significant

First, the in vitro killing speed of SC83288 was determined according to the method developed by Le Manach et al. [56], which classifies drugs as slow- or fast-acting based on a relative in vitro speed of action. To this end, growth inhibition assays were performed, using asynchronous cultures of Dd2, and assessed parasite growth after drug exposure of 24, 48 and 72 h. The IC_50_ ratios were subsequently normalized to the standard 72 h incubation assay. For comparative reasons, artemisinin and atovaquone were analysed in parallel. The IC_50_ ratios of SC82388 were independent of the exposure time (1.28 ± 0.08, 1.24 ± 0.05 and 1.00 ± 0.06 for 24, 48 and 72 h of drug exposure, respectively; One-way ANOVA Holm-Sidak test, p = 0.3), indicating a fast mode of action. Similarly, the exposure time did not affect the IC_50_ ratios of artemisinin (0.97 ± 0.07, 0.88 ± 0.01 and 1.00 ± 0.01 for 24, 48 and 72 h of treatment, respectively; One-way ANOVA Holm-Sidak test, p = 0.2). In comparison, the IC_50_ ratios of atovaquone were significantly different (3.4 ± 0.1, 1.35 ± 0.04 and 1.00 ± 0.04, for 24, 48 and 72 h of treatment, respectively; One-way ANOVA Holm-Sidak test, p < 0.001) (Fig. 5), consistent with atovaquone being a slow acting anti-malarial [56, 57, 60].

**Fig. 6:**
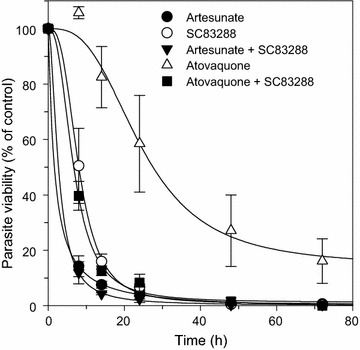
Killing rate profile for artesunate, SC83288, atovaquone, alone and in combination. Asynchronous cultures of Dd2 were exposed to 10-times the IC_50_ concentration of SC82388, artesunate, or atovaquone, alone or in combination, for a duration of 8, 14, 24, 48 or 72 h before the number of re-invading parasites were determined as an indicator of parasite viability. Data were normalized to untreated controls analysed in parallel. Mean ± SEM of four independent determinations

In order to identify possible detrimental interactions between the drugs in the prospect of a potential combination therapy, a second approach was employed to evaluate the absolute speed of action of SC83288, alone as well as in combination with artesunate or atovaquone, based on the method of Linares et al. [57]. To this end, asynchronous cultures of Dd2 were treated with 10x IC_50_ concentrations of SC82388, artesunate or atovaquone, alone or in combination, for a duration of 8, 14, 24, 48 or 72 h. After washing-out the drugs, the parasites were cultured with uninfected erythrocytes stained with a live cells marker, the esterase substrate carboxyfluorescein diacetate (cFDA). After 48 h of subsequent incubation, the DNA of the parasite was stained with Draq5 and the amount of cFDA and Draq5 stained cells was determined by flow cytometry. The results of this assay classified SC83288 as a fast-acting anti-malarial compound, with a 90% parasite clearance time (PCT_90%_) of 17 ± 3 h. In comparison, the measured PCT_90%_ of artesunate and atovaquone were 11 ± 1 h and > 80 h, respectively (Fig. 6). Combining artesunate and SC83288 did not lead to a significant difference in the PCT_90%_ compared with artesunate alone (9.0 ± 0.5 h, Student’s t test, p = 0.1) (Fig. 6). Similarly, combining SC83288 with atovaquone did not significantly alter the PCT_90%_ of SC83288 alone (17 ± 6 h Student’s t test, p = 0.4) (Fig. 6).

## Discussion

Understanding the mode of action and the mechanism of resistance of a therapeutic agent is not mandatory for a compound to be released on the market. However, information regarding these processes may help to predict and control interactions of the compound with the environment and with other drugs deployed in the field. To this purpose, the antiplasmodial action of SC83288 was profiled against artemisinin and artemisinin derivatives in *P. falciparum*.

The parasite reduction ratio after a single dose, i.e. the speed of action of the molecule, is a quality feature of an anti-malarial drug as recently emphasized by the Medicine for Malaria Venture (MMV) [61]. Particularly, drugs against severe malaria are expected to rapidly clear sequestered parasites from the patient´s microvasculature, which can make the difference between survival and death. Moreover, fast-acting anti-malarial drugs are favoured for their ability to reduce the risks of resistant parasites arising within the patient. To this day, the artemisinin derivatives remain the gold standards of a rapidly clearing anti-malarial, reducing the parasite burden within hours [2]. SC83288 has previously been characterized as a fast-acting anti-malarial compound in vitro [20] using a recrudescence assay [60]. Under these conditions, the SC83288 speed of action profile was closer to the fast-acting artemisinin than to the slow-acting atovaquone, although the SC83228 showed a slightly longer clearance time and a lower parasite reduction ratio compared to artemisinin [20]. On a direct clearance-time measurement using trophozoites, SC83288, however, revealed a higher activity than artemisinin [20], consistent with the potent antiplasmodial activity of SC83288 on trophozoites [20]. In this study, the killing speed of SC83288 was verified, using two other methods: a relative speed assay [56], and a re-invasion assay [57]. The results of these assays were consistent with previous observations, marking SC83288 as a fast-acting anti-malarial compound (Figs. 5 and 6), with an in vitro killing speed profile closer to artesunate than to atovaquone (Fig. 6). Further, combining SC83288 with artesunate did not significantly impair the speed of action (Fig. 6). The speed of action is, however, not the only crucial aspect that needs to be taken into account when developing an anti-malarial compound. As for any xenobiotic molecule, the beneficial versus adverse effects must be carefully weighed, and parameters such as safety, pharmacokinetics and drug–drug interactions need to be considered. In this regard, preliminary results on the safety and toxicology of SC83288 in mice and dogs are promising, although a full assessment would await clinical trials in humans. This study further revealed no obvious in vitro interactions between artemisinin and SC83288 in the *P. falciparum* strain Dd2 (Fig. 4). Both the graphical and arithmetical analysis of our isobologram method supported this observation, with the isobologram following a linear trend, and a mean sum of fractional 50% inhibitory concentration falling within the range of an indifferent interaction [55]. An isobologram may overlook possible similar modes of action, particularly if the two tested compounds are both either competitive inhibitors or mutually exclusive non-competitive inhibitors [55]. Competition of both compounds for the same binding site, independently of an interference with the binding site of the endogenous substrate, would not be noticed in an isobologram [55]. However, the chemical and structural differences between SC83288 and the artemisinins (Fig. 1) strongly argue against this possibility.

Resistance is another key aspect of the MMV’s drug candidate profile. Indeed, resistance to most marketed anti-malarial drugs, either previously or still currently used to treat a *P. falciparum* infection, has been identified worldwide to various degrees [62, 63]. Thus, novel drugs need to overcome existing mechanisms of resistance. A previous study has shown that SC83288 is active in vitro against various *P. falciparum* laboratory strains that are resistant to quinoline, quinoline-like and antifolate anti-malarials [20]. This study extends these findings to include the artemisinin resistant parasite strain NF54^ART^ that carry the *kelch 13* mutation associated with reduced artemisinin responsiveness. SC83288 was found to be active against NF54^ART^. Conversely, resistance to SC83288 did not affect the susceptibility of the parasite to other anti-malarial drugs, including artemisinins, chloroquine, mefloquine and the folate antagonists [20].

## Conclusion

While this study does not provide molecular insights into the precise mode of action of SC83288, or into the possible mechanism of resistance, our study suggests that artemisinins and SC83288 target different molecular pathways, based on the absence of cross-reactivity in several independent assays. The absence of cross-resistance and the ability to break established resistances against several anti-malarial drugs, together with a fast in vitro parasite killing speed, are favourable features of SC83288 and would support further development of this compound, possibly as a treatment against severe malaria. Whether SC83288 can be an alternative to artesunate for the treatment of severe malaria, however, has to await clinical safety and efficacy trials in humans.
